# Impact of Insulin Resistance and Preclinical Atherosclerosis Parameters in Long-Term Prediction of Cardiovascular Events: A Seven-Year Prospective Study

**DOI:** 10.3390/jcm15020808

**Published:** 2026-01-19

**Authors:** Daniela Di Lisi, Girolamo Manno, Cristina Madaudo, Francesco Perone, Francesco Leonforte, Antonio Luca Maria Parlati, Andrea Flex, Salvatore Novo, Paolo Tondi, Alfredo Ruggero Galassi, Giuseppina Novo

**Affiliations:** 1 Department of Cardiology, Polyclinic University Hospital Paolo Giaccone, 90127 Palermo, Italy; danydilis@hotmail.it (D.D.L.);; 2 Department of Health Promotion, Mother and Child Care, Internal Medicine and Medical Specialties (ProMISE), University of Palermo, 90127 Palermo, Italy; girolamomanno@hotmail.it; 3 Cardiac Rehabilitation Unit, Rehabilitation Clinic Villa delle Magnolie, Castel Morrone, 81020 Caserta, Italy; 4 Department of Integrated Hygiene, Organizational, and Service Activities (Structural Department), University Hospital Polyclinic G. Rodolico-San Marco, 95123 Catania, Italy; 5 Department of Critical and Rehabilitative Cardiology, Heart Failure and Clinical Cardiology, Centro Cardiologico Monzino, 20138 Milan, Italy; 6 Department of Internal Medicine, Medical Clinic and Vascular Diseases, Fondazione Policlinico Universitario Agostino Gemelli IRCCS, Università Cattolica del Sacro Cuore, 00168 Rome, Italy; 7 Division of Angiology, Fondazione Policlinico Universitario Agostino Gemelli IRCCS, L.go A. Gemelli 8, 00168 Rome, Italy

**Keywords:** atherosclerosis, insulin resistance, metabolic syndrome, cardiovascular risk factors, preclinical markers, arterial stiffness, speckle-tracking echocardiography, preventive cardiology, risk prediction

## Abstract

**Background/Objectives**: Cardiovascular (CV) and cerebrovascular diseases, primarily attributed to atherosclerosis, stand as leading global causes of morbidity and mortality. This study aims to evaluate the impact of preclinical atherosclerosis parameters, including intima-media thickness (IMT) and arterial stiffness, in a seven-year follow-up of 100 patients with CV risk factors but no known history of CV or cerebrovascular diseases. **Methods**: Between April 2014 and December 2015, 100 patients presenting with suspected ischemic heart disease were enrolled. The study integrates the color Doppler examination of the supra-aortic trunks with the evaluation of preclinical parameters of atherosclerosis, such as intima-media thickness (IMT), βeta index, and pulse wave velocity (PWV), as well as echocardiographic evaluations, including global longitudinal strain (GLS). CV risk factors, metabolic syndrome, and insulin resistance were assessed and measured for each patient using the Homeostasis Model Assessment of Insulin Resistance (HOMA-IR). Two- and seven-year follow-ups assessed various CV events. **Results**: The study population comprised 67% males and 33% females. Metabolic syndrome, impaired fasting glycemia and hypertension were prevalent. The mean value of IMT was 1.21 ± 0.26 mm, and PWV was 8.47 ± 2.14 m/s. The 7-year follow-up identified IMT, PWV, and HOMA-IR as strong positive predictors of cardiovascular events, with PWV emerging as a particularly sensitive indicator of early events. **Conclusions**: Insulin resistance and cardiovascular risk factors may contribute to early alterations in myocardial and vascular function, even in the absence of overt disease. PWV, as a recognized surrogate marker of arterial stiffness, may serve as a sensitive tool for the early prediction of cardiovascular events. A comprehensive screening, including the assessment of markers indicating subclinical vascular alterations, along with the implementation of preventive interventions, is crucial for populations at risk.

## 1. Introduction

Cardiovascular (CV) and cerebrovascular diseases are the leading cause of mortality and morbidity worldwide [1]. Atherosclerosis is recognized as the main underlying cause of cardiovascular diseases [2]. Atherosclerosis is a pathological process with a complex evolution that tends to develop over time in an entirely asymptomatic manner [3]. Symptoms manifest during the advanced stages of the process, making clinical management challenging. Clinical manifestations are sometimes fatal, such as acute myocardial infarction, cerebral ischemic stroke and critical ischemia of the lower limbs [4]. An effective treatment strategy involves identifying and correcting CV risk factors by implementing primary prevention measures. In a study by Mackinnon et al., the close correlation between cardiovascular risk factors for atherosclerosis and the specific localization of subclinical atherosclerotic lesions within the arterial wall was evaluated [5]. Equally useful could be the identification and monitoring of markers of preclinical atherosclerosis, which can detect early structural vascular changes that may predispose the subject to the development of clinically manifest atherosclerotic disease [6]. As is well known, intima-media thickness (IMT) serves as an early marker of subclinical atherosclerosis affecting multiple vascular territories [7]. The correlation between this parameter, metabolic syndrome and global CV risk is well recognized [8,9,10]. Although the measurement of IMT can reveal early vascular changes, it reflects organic damage that is already established and present at a systemic level.

The evaluation of arterial stiffness reflects an earlier functional alteration that may precede structural changes, such as increased IMT [11]. The βeta index of carotid arterial stiffness has been studied in the context of cardiovascular diseases and correlated as an index of risk of myocardial infarction [11,12], aortic stenosis [13], metabolic syndrome [14] and cardiotoxicity [15]. Pulse wave velocity (PWV) is now considered the simplest, most valid and reproducible parameter for determining regional arterial stiffness [16]. PWV was also used in cardio-oncology to detect early subclinical vascular damage [15]. A linear correlation exists between the speed and stiffness with which a wave travels along an arterial segment. Carotid-femoral PWV is considered the gold standard for measuring arterial stiffness [17] and is used in most epidemiological studies which have demonstrated a substantial predictive value as an independent factor for cardiovascular events [18]. In the literature, several studies have shown the importance of evaluating arterial stiffness as a more sensitive parameter than IMT for evaluating vascular pathology [19,20]. Our study aimed to assess the impact of preclinical parameters of atherosclerosis over a seven-year follow-up in healthy subjects without a prior CV clinical history but with CV risk factors.

## 2. Materials and Methods

All consecutive patients admitted to our center with suspected ischemic heart disease between April 2014 and December 2015 were prospectively enrolled in the study. Each patient underwent coronary angiography (CAG), transthoracic echocardiography, and color Doppler ultrasound of the supra-aortic trunks. Inclusion criteria were as follows: patients with one or more cardiovascular risk factors and suspected coronary artery disease (CAD) but no obstructive CAD on coronary angiography. Specifically, this included patients with either entirely normal coronary arteries (0% stenosis) or non-significant luminal irregularities, defined as <30% stenosis and not associated with ischemia. The exclusion criteria were as follows: obstructive CAD highlighted on coronary angiography or presence of intramyocardial bridges, the elevation of markers of myocardial necrosis, diabetes mellitus (DM) (fasting blood glucose greater than or equal to 126 mg/dL or treatment with insulin or other hypoglycemic drugs oral), creatinine greater than or equal to 1.5 mg/dL, left ventricular hypertrophy (wall thickness on echocardiography > 12 mm), more than mild valvular pathologies, atrial fibrillation or other arrhythmias interfering with ECG-graphic triggering, ejection fraction less than 50% and the absence of carotid artery stenosis at baseline.

The following CV risk factors were evaluated: arterial hypertension (>130/85 mmHg), obesity (Body Mass Index, BMI > 30 kg/m^2^ or abdominal circumference > 102 cm in males or >88 cm in females), dyslipidemia (hypertriglyceridemia > 150 mg/dL, or HDL cholesterol < 40 mg/dL in males or <50 mg/dL in females, LDL cholesterol >130 mg/dL), cigarette smoking, family history of CV disease, impaired fasting glycaemia (fasting glycaemia > 100 mg/dL) or impaired tolerance to carbohydrates (glycaemia after 2 h after a meal >140 mg/dL). Insulin resistance (IR) was objectively measured by measuring the Homeostasis Model Assessment of Insulin Resistance (HOMA-IR = Fasting blood sugar × Insulinemia/405), a surrogate parameter of insulin resistance, which relates glycemia and basal insulinemia, according to the formula proposed by Matthews et al. [21]. According to literature data, a diagnosis of IR was made when HOMA-IR values were higher than 2 [22]. Patients underwent two venous blood tests at enrollment to determine glycemia and insulin levels. The first was at 8:00 AM, 12 h after fasting, and the second at 10:00 AM, 2 h after the meal. Blood glucose samples were analyzed within an hour of testing sampling using colorimetric tests (GLU vitro slides). In contrast, samples for insulin dosing were stored at −20 °C and subsequently analyzed with the ELISA method with a specific kit (Insulin ELISA test kit, DIAMETRA, ALIFAX s.r.l, Padua, Italy, code DKO076). Hemolyzed samples were excluded.

### 2.1. Echocardiography and Supra-Aortic Trunks Color Doppler Examinations

Myocardial damage was assessed using the echocardiographic method, and subclinical and early alterations in longitudinal systolic function were looked for using tissue Doppler and speckle tracking echocardiography (STE). The echocardiographic examination was performed with a “Siemens ACUSON SC200 PRIME Ultrasound System (Siemens Healthineers, Erlangen, Germany)”. The echocardiographic evaluation included the acquisition of the four standard projections (parasternal long axis, parasternal short axis, apical 4-, 2-, and 3-chamber views). The left ventricular ejection fraction (LVEF) was calculated according to the EACVI/ASE Guidelines, using the Simpson Biplane method from the apical 4-chamber to apical 2-chamber projections [23]. All echocardiographic evaluations included those required by the EACVI/ASE recommendations [24]. Global longitudinal strain (GLS) was measured using STE, and the evaluations were conducted offline on previously acquired routine two-dimensional clips, with a frame rate set between 60 and 110 Hz [23]. The VVI (Velocity Vector Imaging) with 5.1 Software was used to measure the STE, supplied with a “Siemens ACUSON SC200 ultrasound system”. All patients examined were in sinus cardiac rhythm. A GLS value of −19.8 ± 4.6% was considered normal [25]. Two echocardiographers performed the measurements, unaware of the patient’s risk factors and HOMA-IR values. The average value between the two determinations was considered.

Vascular damage was studied using Doppler ultrasonography of the supra-aortic trunks. An Esaote MyLabA70^TM^ ultrasound machine (Esaote S.p.A., Genoa, Italy) was used. Carotid stiffness was measured using “Esaote QAS” software v.2.1.3 (Esaote S.p.A., Genoa, Italy). Intima-media thickness (IMT, mm) and carotid stiffness, measured in terms of the βeta index (a dimensional logarithmic index) and pulse wave velocity (PWV, expressed in m/s), were assessed to evaluate preclinical atherosclerosis. M-mode ultrasound of the common carotid artery bulb was performed using a 7.5 MHz high-resolution probe to measure IMT. Following the ESH-ESC (European Society of Hypertension, European Society of Cardiology) guidelines [17,26], IMT was considered:

- Normal if <0.9 mm;
- Increased if between 0.9 and 1.5 mm;
- Indicative of asymptomatic carotid plaque (ACP) if >1.5 mm;

and the value of 6.65 m/s of PWV was considered the normal cut-off [27]. For both the IMT and the arterial stiffness parameters, the highest value measured at the right and left carotid levels was considered for each patient, as it was indicative of a greater risk for cardiovascular events. The exams were performed by two blind operators using the average of the measurements.

### 2.2. Follow-Ups and Outcomes

**Figure 1:**
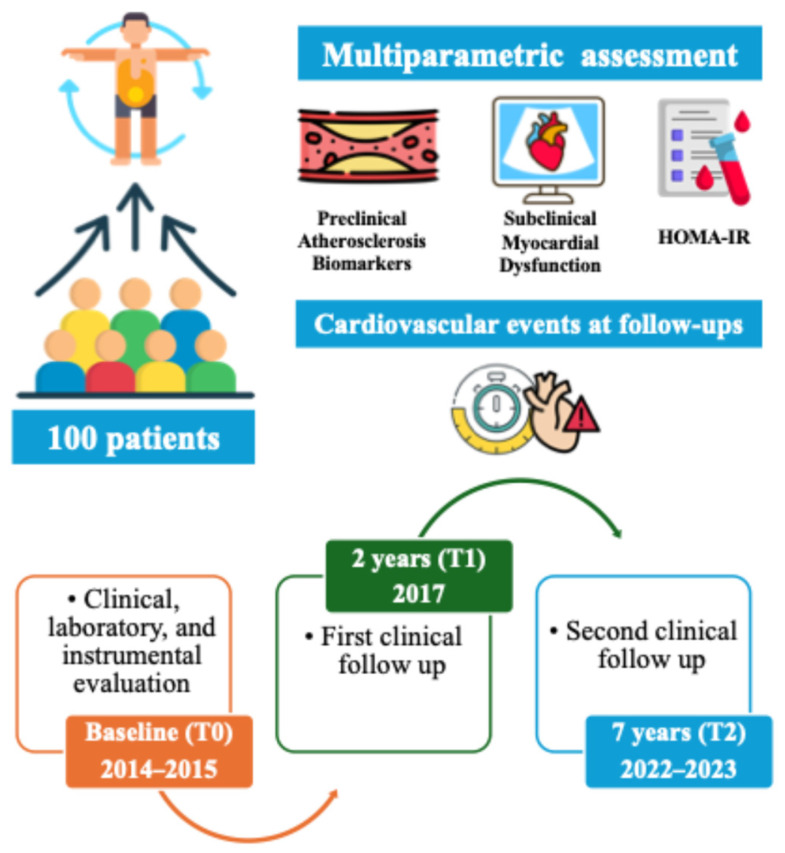
Timeline of the research work.

A first telephone follow-up was conducted in 2017 (T1), and a second clinical follow-up was performed in 2022 and 2023 (T2). The timeline of the research work is shown in Figure 1. All recruited patients who survived completed the scheduled assessments. Clinical events were evaluated in two distinct time intervals: from baseline to the first follow-up in 2017 (T1), and from T1 to the second follow-up in 2022/2023 (T2). The median follow-up time was 2 years (interquartile range [IQR] 1.9–3.0) for the 2-year (T1) model and 7.3 years (IQR 6.9–7.7) for the 7-year (T2) model. The 2-year follow-up (T1) was chosen to capture short-term metabolic and functional outcomes, while the 7-year evaluation (T2) aimed to assess long-term atherosclerotic and ischemic events. This design reflects the progressive nature of cardiometabolic disease, where early metabolic alterations precede overt vascular events.

The events recorded in the follow-ups were as follows: diagnosis of diabetes mellitus, paroxysmal atrial fibrillation; transient ischemic attack (TIA), stroke (minor and major stroke), unstable angina, acute myocardial infarction with persistent ST-segment elevation (STEMI), acute myocardial infarction without persistent ST-segment elevation (NSTEMI), peripheral arterial disease (PAD), carotid atherosclerosis (defined as the presence of atherosclerotic plaques causing ≥30% luminal narrowing on Doppler ultrasound of the supra-aortic trunks), CV mortality, and all-cause mortality.

### 2.3. Statistical Analysis

Data were expressed as mean ± standard deviation (SD) for continuous variables, or as percentages for categorical variables. The distribution of continuous variables was assessed using the Shapiro–Wilk test. In the absence of a normal distribution, data were presented as median and interquartile range (IQR). For comparing binary outcomes measured at two different time points in the same individuals, McNemar’s test was applied to account for the paired nature of the data. During follow-up, more than one cardiovascular or cerebrovascular event could occur in the same individual (e.g., diabetes onset, atrial fibrillation, transient ischemic attack). Therefore, descriptive tables report total event counts, whereas in time-to-event Cox regression analyses only the first event per patient was considered for the composite endpoint. Time-to-event analyses were performed using univariable and cause-specific multivariable Cox proportional hazards models. The exact timing of each event was available, allowing proper survival analysis. Two separate models were constructed: one for T1 events (occurring within 2 years of follow-up) and one for T2 events (occurring within 7 years). Clinically relevant variables with less than 5% missing data and a *p*-value ≤ 0.05 in univariable analysis were included in the multivariable model using a full-model approach to identify independent predictors of myocardial dysfunction and arterial stiffness. A *p*-value < 0.05 was considered statistically significant. Statistical analyses were performed using RStudio software, version 2024.12.1+563 (Posit PBC, Boston, MA, USA)

## 3. Results

The study population consists of 100 patients (Male = 67%, Female = 33%). The average age of the patients was 62.9 ± 9.45 years, with a body mass index (BMI) of 28.50 ± 2.71 kg/m^2^. Sixty-five patients (65%) had a diagnosis of metabolic syndrome. The main risk factor was impaired fasting glycemia (69%), with an average fasting glycemia value, measured at 8:00 AM, of 104.96 ± 11.08 mg/dL. At the same time, the corresponding basal insulinemia value equals 17.63 ± 9.08 µU/mL. The average HOMA-IR index was 4.84 ± 2.56. Forty-six patients (46%) were hypertensive. All hypertensive patients were under pharmacological treatment, with systolic blood pressure values of 132.19 ± 11.24 mmHg and diastolic blood pressure values of 85.60 ± 8.31 mmHg. ACE-I and beta-blockers were the most widely used pharmacological treatments (25% of the population, respectively). We found that 57% of the population had dyslipidemia (of which 30% had hypertriglyceridemia and 27% reduced LDL). The clinical and laboratory parameters of the study population are summarized in Table 1.

Regarding the parameters relating to preclinical atherosclerosis, IMT was 1.21 ± 0.26 mm, while the βeta index was 12.19 ± 5.44 and the PWV was 8.47 ± 2.14 m/s. Other ultrasonographic parameters of the study population were reported in Table 2.

The most frequent events recorded in the two-year follow-up (T1) were the diagnosis of type II diabetes mellitus in 18 patients (18%) and paroxysmal atrial fibrillation in 28 patients (28%). The same trend was also repeated in the seven-year follow-up (T2), with a considerable increase in TIA cases found in 18 patients (18%) and carotid atherosclerosis in 13 patients (13%). Table 3 presents the main events recorded during the two follow-ups.

Multiple events per patient were common. Overall, 53 patients (53%) experienced at least one cardiovascular or cerebrovascular event at the 2-year follow-up (T1) as first event and 57 patients (57%) at the 7-year follow-up (T2). The apparent high cumulative number of events (Table 3) reflects the fact that some individuals experienced more than one outcome during follow-up. In univariate analysis, at T1 and T2, both IMT, βeta index and PWV were positive predictors of events. In the multivariable model, only the PWV parameter was statistically significant at T1 (Odds Ratio 2.01, 95% CI 1.21–3.33, *p*-value = 0.007) (Table 4). At T2, multivariate analysis revealed that both IMT and PWV are independent predictive variables of CV events (IMT Odds Ratio 1.16, 95% CI 1.02–1.32, *p*-value = 0.02; PWV Odds Ratio 1.38, 95% CI 1.10–1.74, *p*-value = 0.006) (Table 4). HOMA-IR was also a strong independent predictor of CV events at 2- and 7-year follow-ups (Table 4).

## 4. Discussion

Insulin resistance and CV risk factors lead to significant subclinical alterations at the myocardial level, as evaluated by a reduction in GLS, and at the vascular level, estimated by an increase in IMT and arterial stiffness [14]. However, to our knowledge, our study was the first to conduct a multiparametric evaluation, including preclinical atherosclerosis parameters (IMT, arterial stiffness), subclinical myocardial damage parameters (expressed in terms of GLS), and HOMA-IR. HOMA<-IR is a simple powerful marker of insulin resistance to be evaluated in clinical practice. This study demonstrated the importance of the IMT parameter, PWV and HOMA-IR as strong positive predictive factors for cardiovascular events 7 years after enrollment. Interestingly, the arterial stiffness parameter PWV is a positive predictive factor of early CV events, proving even more sensitive from the first follow-up in identifying subclinical vascular damage. Furthermore, patients who presented subclinical alterations at baseline, especially vascular ones, had a higher incidence of development of cardiovascular and cerebrovascular events and an increase in mortality from cardiovascular causes. Ikonomidis et al. observed that insulin resistance and acute changes in glucose levels can lead to endothelial dysfunction, resulting in increased stiffness and reduced coronary flow reserve [28]. In the SEARCH CVD study, the variation in arterial stiffness, as measured by PWV, was evaluated in 298 young individuals with type 1 diabetes mellitus.

The reduced insulin sensitivity in these subjects represented a genuine, independent cardiovascular risk factor, leading to early arterial stiffness [29]. Another study also confirmed the close relationship between insulin resistance and arterial stiffness, showing a significant and direct correlation between HOMA-IR and PWV in normotensive HCV^+^ patients, in which stiffness reaches values similar to those of hypertensive patients [30]. Another interesting data source in our work was HOMA-IR, which was identified as a strong independent predictive factor of CV events and remained significant at both the 2-year and 7-year follow-ups. Insulin resistance can contribute to ventricular and vascular dysfunction through several direct and indirect mechanisms [31]. It directly promotes the increased deposition of extracellular matrix and collagen, as well as the reduction of degradation mechanisms (through the downregulation of MMPs and upregulation of TIMPs) [32]. It causes alterations in fatty acid metabolism and, secondarily, inhibits glucose metabolism.

Furthermore, patients with insulin resistance can present a real “cluster” of cardiovascular risk factors, resulting in metabolic syndrome [33,34]. For this reason, we enrolled patients who did not have conditions that could influence left ventricular and vascular function, such as myocardial ischemia, ventricular hypertrophy, and renal disease, to avoid the effects of confounding factors and to analyze the direct effects of insulin resistance effectively. Insulin resistance also has both indirect and direct impacts on the vascular system. Among the direct ones, endothelial damage and dysfunction lead to impairment of the vasomotor response to the main endothelium-dependent vasodilatory stimuli, as well as pro-inflammatory and pro-coagulant activity of the endothelium [7,31]. Indirect mechanisms, however, act together with cardiovascular risk factors. Therefore, HOMA-IR may have a strong prognostic value for cardiovascular events, and its broader use could be beneficial in clinical practice [35]. Moreover, the application of artificial intelligence techniques to multiparametric cardiovascular data may further improve risk stratification by identifying complex interactions among metabolic, vascular, and myocardial parameters that are not easily captured through traditional statistical methods [36,37]. In this regard, larger prospective cohort studies and real-world data analyses could provide additional insights, supporting the development of more accurate and personalized predictive models. These parameters (HOMA-IR, PWV, IMT) could be used for primary prevention in patients at high risk of CAD. SCORE2 and ASCVD remain the main scores to be used in clinical practice in accordance with the current guidelines but the assessment of arterial stiffness, global longitudinal strain, IMT and HOMA-IR could provide more prognostic information, also allowing the identification of subclinical and early vascular damage. Thus, an integrated approach using traditional scores and HOMA-IR, PWV is desirable in clinical practice. More studies are needed to confirm the added value of these parameters.

## 5. Limitations of the Study

Considering the low incidence of cardiovascular events, we have considered all the events together. This study was an exploratory single-center cohort of 100 consecutively enrolled patients, designed to evaluate the long-term prognostic role of subclinical vascular and metabolic markers rather than to provide definitive effect estimates. Among other limitations of the present study, we must acknowledge the relatively small sample size. However, the application of strict exclusion criteria allowed us to obtain a well-defined and representative cohort, minimizing potential confounding factors and reducing bias. Another limitation is the lack of follow-up monitoring for instrumental and laboratory parameters, including inflammatory markers such as C-reactive protein (CRP), which were not available for the study population. Furthermore, the SARS-CoV-2 pandemic significantly impacted data collection, hindering the continuation of patient assessments during the follow-up period.

Another limitation of the study was the low events-per-variable ratio that made the multivariate analysis prone to overfitting.

## 6. Conclusions

The results of our study demonstrated a correlation between CV risk factors and insulin resistance and subclinical alterations at the vascular level, as indicated by markers of preclinical atherosclerosis, including IMT and increased arterial stiffness. Patients with insulin resistance, even without diabetes mellitus, CAD and hypertensive heart disease, represent a population at increased risk. These patients should undergo careful screening programs, lifestyle modifications, and possibly targeted medications to avoid the onset of myocardial and vascular damage. Furthermore, our results underline the importance of studying the close correlation between myocardial and vascular-arterial function to use, if necessary, preventive and therapeutic interventions for both pathologies. Ultrasound methods effectively identify subclinical dysfunction early as a predictor of CV events. For this reason, its use is recommended in selected categories of patients considered to be at high risk. However, more extensive studies will be necessary in the future to confirm these findings.

## Figures and Tables

**Table 1:** Clinical and Laboratory Parameters of the Study Population at baseline (T0).

Parameters	
Age (years)	62.9 ± 9.45
Men sex	67 (67)
BMI (kg/m^2^)	28.50 ± 2.71
Hypertriglyceridemia	30 (30)
Reduced HDL	16 (16)
Elevated LDL	27 (27)
Hypertension	46 (46)
Impaired Fasting Glucose	69 (69)
Increased waist circumference	24 (24)
Metabolic Syndrome	65 (65)
Family history of cardiovascular disease	60 (60)
Smoking History	35 (35)
Systolic Blood Pressure (mmHg)	132.19 ± 11.24
Diastolic Blood Pressure (mmHg)	85.60 ± 8.31
Fasting Blood Sugar at 8 AM (mg/dL)	104.96 ± 11.08
Insulin Level at 8 AM (µU/mL)	17.63 ± 9.08
Fasting Blood Sugar at 10 AM (mg/dL)	124.32 ± 19.78
Insulin Level at 10 AM (µU/mL)	33.89 ± 15.49
HOMA-IR	4.84 ± 2.56

Values are reported as mean ± SD and n (%). BMI: Body Mass Index, HDL: High-Density Lipoprotein, LDL: Low-Density Lipoprotein.

**Table 2:** Ultrasonographic Parameters of the Study Population.

Parameters	
IVS (mm)	11.1 ± 1.12
LVEDD (mm)	44.43 ± 5.82
LVEDV (mL)	97.67 ± 2.84
LVEF (%)	55.67 ± 6.56
LAVi (mL/m^2^)	31.43 ± 8.56
E/A	0.94 ± 0.35
DT (ms)	234.94 ± 57.14
S’-TDI (cm/s)	7.56 ± 1.12
e’-TDI (cm/s)	8.43 ± 1.87
a’-TDI (cm/s)	10.12 ± 2.16
E/e’ ratio	7.83 ± 1.27
sPAP (mmHg)	26.77 ± 5.41
TAPSE (mm)	23.21 ± 3.24
LV GLS (%)	−18.68 ± 2.52
IMT (mm)	1.21 ± 0.26
βeta index	12.19 ± 5.44
PWV (m/s)	8.47 ± 2.14

Values are reported as mean ± SD and n (%). IVS: Interventricular Septum, E/A: E wave (early Diastole)/A wave (late diastole), EDD: End-Diastolic Diameter, EDV: End-Diastolic Volume, EF: Ejection Fraction, LAVi: Left Atrial Volume indexed to Body Surface Area, LV: left ventricle, DT: Deceleration Time, sPAP: Systolic Pulmonary Arterial Pressure, TAPSE: Tricuspid Annular Plane Systolic Excursion, TDI: Tissue Doppler imaging, S’: systolic wave velocity, e’: early diastolic mitral annular velocity, a’: late diastolic mitral annular velocity, E/e’ ratio: E wave (early Diastole)/early diastolic mitral annular velocity, GLS: Global Longitudinal Strain, IMT: Intima-Media Thickness, PWV: Pulse Wave Velocity.

**Table 3:** Events recorded in the clinical follow-up.

Events	2017	2022–2023	*p*-Value
T2DM	18% (18)	37% (37)	0.002
Paroxysmal AF	28% (28)	34% (34)	0.3
TIA	4% (4)	18% (18)	0.001
Minor Stroke	1% (1)	10% (10)	0.005
Major Stroke	1% (1)	10% (10)	0.005
Unstable Angina	1% (1)	7% (7)	0.030
NSTEMI	1% (1)	11% (11)	0.002
STEMI	0% (0)	1% (1)	0.3
PAD	1% (1)	11% (11)	0.002
Carotid ATS	0% (0)	13% (13)	0.0002
CV Death	0% (0)	7% (7)	0.007
All-Causes Death	0% (0)	1% (1)	0.03

Values are reported n (%). T2DM: Type II Diabetes Mellitus, AF: Atrial Fibrillation, TIA: Transient Ischemic Attack, NSTEMI: Non-ST-Elevation Myocardial Infarction, STEMI: ST-Segment Elevation Myocardial Infarction, PAD: Peripheral Artery Disease, ATS: Atherosclerosis, CV: Cardiovascular.

**Table 4:** Univariable and Multivariable Cox Proportional Hazards Models for Predictors of T1 (2-year) and T2 (7-year) Events in the Overall Population.

	Overall Population n = 100					
	Univariate Analysis			Multivariate Analysis		
**2 Years Events (T1)**	HR	95% CI	*p* value	HR	95% CI	*p* Value
Age	1.02	0.94, 1.10	0.7			
Elevated Triglycerides	0.36	0.04, 2.98	0.34			
Elevated LDL	4.25	0.95, 2.98	**0.05**	2.50	0.37, 17.1	0.3
Arterial Hypertension	0.85	0.19, 3.82	0.84			
Smoke	0.83	0.16, 4.30	0.83			
Obesity (BMI > 30)	6.67	1.47, 30.3	**0.014**	0.99	0.10, 9.94	ns
Family history of CVD	0.89	0.20, 3.99	0.88			
LVEF	0.90	0.75, 1.08	0.25			
LV GLS	2.12	1.15, 3.90	**0.016**	0.84	0.33, 2.18	0.7
IMT (mm)	1.39	1.01, 1.92	**0.04**	1.21	0.86, 1.59	0.3
βeta index	1.03	0.92, 1.15	0.65			
PWV (m/s)	1.43	1.06, 1.93	**0.01**	2.01	1.21, 3.33	**0.007**
HOMA-IR	1.87	1.27, 2.76	**0.002**	2.09	0.98, 4.47	**0.041**
**7 years events (T2)**	HR	95% CI	*p* value	HR	95% CI	*p* value
Age	1	0.97, 1.04	0.83			
Elevated Triglycerides	0.56	0.27, 1.16	0.12			
Elevated LDL	1.94	1.04, 3.62	**0.037**	0.99	0.36, 1.74	0.6
Arterial Hypertension	1.36	0.74, 2.49	0.32			
Smoke	1.72	0.94, 3.16	0.08			
Obesity (BMI > 30)	3.29	1.78, 6.10	**<0.001**	1.75	0.77, 4.01	0.2
Family history of CVD	0.53	0.29, 0.97	0.05			
LVEF	0.93	0.87, 1.00	0.05			
LV GLS	1.60	1.33, 1.93	**<0.001**	1.20	0.90, 1.60	0.2
IMT (mm)	1.26	1.12, 1.43	**<0.001**	1.16	1.02, 1.32	**0.02**
βeta index	1.06	1.02, 1.11	**0.004**	1.01	0.94, 1.10	0.7
PWV (m/s)	1.36	1.18, 1.55	**<0.001**	1.38	1.10, 1.74	**0.006**
HOMA-IR	1.49	1.32, 1.68	**<0.001**	1.50	1.32, 1.68	**0.001**

BMI: Body Mass Index, CVD: Cardiovascular Disease, EF: Ejection Fraction, LDL: Low-Density Lipoprotein , LV: left ventricle, GLS: Global Longitudinal Strain, IMT: Intima-Media Thickness, PWV: Pulse Wave Velocity.

## Data Availability

Data will be available upon reasonable request.

## Abbreviations

The following abbreviations are used in this manuscript:

ACE	Angiotensin-converting enzyme
ACP	asymptomatic carotid plaque
ATS	atherosclerosis
BMI	Body Mass Index
CV	Cardiovascular
CAD	coronary artery disease
ESH	European Society of Hypertension
ESC	European Society of Cardiology
GLS	Global longitudinal strain
HDL	high-density lipoprotein
HOMA-IR	Homeostasis Model Assessment of Insulin Resistance
IMT	Intima-media thickness
IQR	interquartile range
IR	Insulin resistance
LDL	low-density lipoprotein
LVEF	left ventricular ejection fraction
NSTEMI	Non-ST-segment elevation myocardial infarction
PAD	Peripheral arterial disease
PWV	Pulse Wave Velocity
SD	Standard Deviation
STE	Speckle Tracking Echocardiography
STEMI	ST-segment elevation myocardial infarction
TIA	Transient ischemic attack
VVI	Velocity Vector Imaging
